# Removal of Hexamethyldisiloxane via a Novel Hydrophobic (3–Aminopropyl)Trimethoxysilane-Modified Activated Porous Carbon

**DOI:** 10.3390/molecules28186493

**Published:** 2023-09-07

**Authors:** Siqi Lv, Yingrun Wang, Yanhui Zheng, Zichuan Ma

**Affiliations:** 1 Hebei Key Laboratory of Inorganic Nano-Materials, College of Chemistry and Material Sciences, Hebei Normal University, Shijiazhuang 050024, China; lvsq@stu.hebtu.edu.cn (S.L.); yrwang00@stu.hebtu.edu.cn (Y.W.); 2 College of Chemical Technology, Shijiazhuang University, Shijiazhuang 050035, China

**Keywords:** activated porous carbon, APTMS, hydrophobic, hexamethyldisiloxane, adsorption

## Abstract

Volatile methyl siloxanes (VMS) must be removed because the formation of silica in the combustion process seriously affects the resource utilization of biogas. Herein, a series of APTMS ((3–aminopropyl)trimethoxysilane)-modified activated porous carbon (APC) adsorbents (named APTMS@APC) were prepared for VMS efficient removal. The as-prepared adsorbents were characterized using SEM, FTIR, Raman, X-ray diffraction analyses, and N_2_ adsorption/desorption. The results showed that the surface modification with APTMS enhanced the hydrophobicity of APC with the water contact angle increasing from 74.3° (hydrophilic) to 127.1° (hydrophobic), and meanwhile improved its texture properties with the *S*_BET_ increasing from 981 to 1274 m^2^ g^−1^. The maximum breakthrough adsorption capacity of APTMS@APC for hexamethyldisiloxane (L2, model pollutant) was 360.1 mg g^−1^. Effects of an inlet L2 concentration (31.04–83.82 mg L^−1^) and a bed temperature (0–50 °C) on the removal of L2 were investigated. Meanwhile, after five adsorption–desorption cycles, the APTMS@APC demonstrated a superior cycling performance. This indicated that the hydrophobic APTMS@APC has a great significance to remove VMS.

## 1. Introduction

Biogas, a green, ecofriendly, and valuable renewable biofuel, is one of the alternatives of fossil gasoline [1]. Traditionally, biogas is generated at sewage treatment plants and landfills, with methane constituting more than 70% (percent vol.) [2]. In addition to containing methane, biogas comprises some other components, such as carbon dioxide (CO_2_, 30–50 percent vol.), hydrogen sulfide (H_2_S, 5000–10,000 ppm), ammonia (NH_3_, 100 ppm), and volatile methyl siloxanes (VMS, traces, 3–150 mg m^−3^) [3,4,5]. With the improvement of urbanization, silicone materials are widely used, and more than 90% of all the VMS entering the environment end up in the atmosphere [6]. Recent research claims that VMS should be classified as an emerging environmental pollutant [6,7]. In particular, the presence of VMS will be converted to microcrystalline silica during combustion [8] (Equation (1)), which will impair the functions of generator components, lubricating oil and fuel catalysts, thus affecting the energy utilization of biogas [9,10]. Therefore, there is an urgent need to remove VMS before biogas can be used.

((CH_3_)_2_SiO)_n_ + 4n O_2_ → n SiO_2_ + 2n CO_2_ + 3n H_2_O (1)

Several techniques have been used to remove VMS from biogas, including cryogenic condensation, biological technology, catalytic processes, membrane separation, absorption, and adsorption [8,11,12,13,14,15,16]. Among these technologies, physical adsorption through porous materials is considered to be very promising for VMS removal, due to its low cost, ease of operation, high efficiency, and great versatility [17,18]. In the past several years, many porous absorbent materials have been developed and thoroughly investigated, such as activated porous carbon materials, silica gel, molecular sieves, and reduced graphene oxide aerogel (rGOA), with properties compared in Table S1 [14,15,17,19,20,21,22,23,24]. Based on the previous review, despite the large specific surface area and highly developed porous structure, the recycling performance of activated porous carbon materials, silica gel, and molecular sieves is limited, owing to the presence of polar functional groups [25,26,27]. On the contrary, rGOA has a strong hydrophobicity and good recycling performance, but a small specific surface area causes it to have a weak VMS adsorption capacity [28,29]. Therefore, this study aims to develop a novel material with high large specific surface area and hydrophobicity to improve the purification effect of VMS.

Activated porous carbon (APC) with hydrophobic nature is a promising solution. In our recently published paper, we prepared a NaOH-activated porous carbon, which had a large specific surface area and an excellent adsorption property for L2 [14]. However, in the activation process, NaOH would corrode the glass tube, which resulted in higher requirements on the equipment. As reported, H_3_PO_4_-activated carbon could simultaneously possess large surface area and ultra-high pore volume, but with poor hydrophobicity [30,31]. In order to solve this problem, researchers normally employ oxidation treatment, acid–base surface modification, and hydrophobic coating in an attempt to improve the surface hydrophobicity of APC [32]. The former two methods are usually deemed to be laborious with limited enhancement in the hydrophobization of APC [33]. Low surface-energy materials have been used for surface grafting, e.g., silane solutions, Teflon, and other moisture-repellent polymeric materials, to enhance the surface hydrophobicity [33]. Liu et al. synthesized a series of hydrophobic-modified (polydimethylsiloxane (PDMS) coating)-activated carbons (AC) by depositing PDMS on AC using a thermal evaporation method [32]. It can be found that the PDMS coating could induce a change in the surface chemistry, producing a more hydrophobic surface, but the BET surface area had a slight reduction. Therefore, there is an urgent need to find a new hydrophobic coating reagent which can not only meet the hydrophobicity enhancement, but also improve the pore structure and BET surface area.

**Figure 1:**
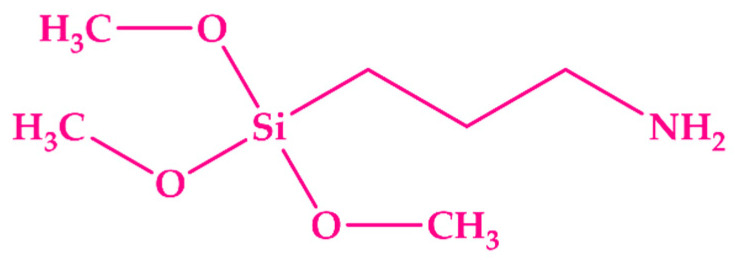
Structural formula of APTMS.

Recently, we are more concerned about (3–aminopropyl)trimethoxysilane (APTMS), as shown in Figure 1, which can not only easily be grafted to APC and enhance the hydrophobization of the materials, but also improve the surface performance due to the amine functional group; and we expect that the enhanced hydrophobization, combined with their textural properties of the as-synthesized APTMS@APC, will provide an efficient removal of VMS from biogas. To demonstrate this possibility, herein, APTMS@APC–x (x is the mass fraction of APTMS) were prepared via APTMS as a grafting agent, H_3_PO_4_ as an activating agent, and coconut shell waste as an inexpensive raw material. Moreover, the hydrophobic coating of APC is successfully prepared in a water dispersion of APTMS, which is easy to operate and is environmentally friendly. It is worth mentioning that after coating, the specific surface area and pore volume do not decrease but slightly increase. In a fixed-bed dynamic adsorption unit, the effect of the prepared adsorbents on the volatile hexamethyldisiloxane (L2, a model compound) removal performances were evaluated. Furthermore, the structure–activity relationship, the influential factors on the adsorption capacity, and the adsorption–desorption tests were also carried out on the best APTMS@APC–x adsorbent. The results proved that the APTMS@APC with both high hydrophobicity and total micropore volume, had a good potential for VMS removal.

## 2. Results and Discussion

### 2.1. Basic Characterization of APC and APTMS@APC–0.125

**Figure 2:**
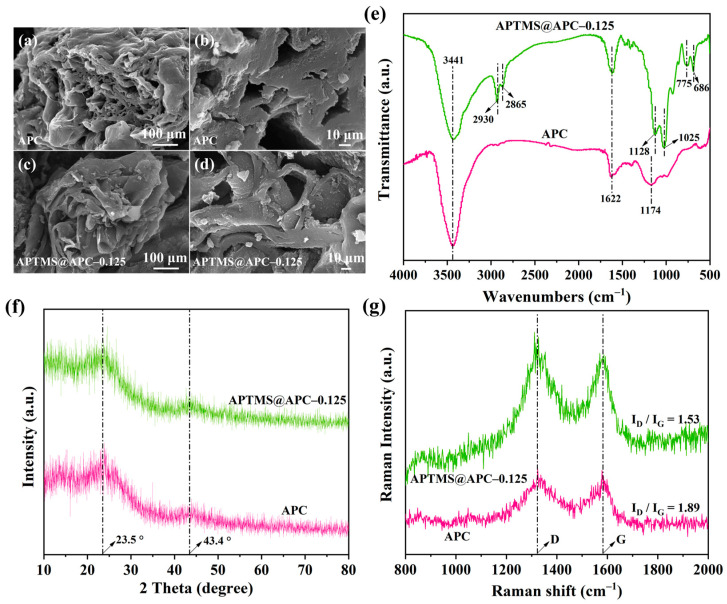
SEM images (**a**–**d**), FTIR spectra (**e**), XRD patterns (**f**), and Raman spectra (**g**) of APC and APTMS@APC–0.125.

Figure 2a–d show SEM images of APC and APTMS@APC–0.125. The SEM diagrams provide interesting morphologic information about the adsorbent. It can be noted that the porosity of APC and APTMS@APC–0.125 was observed. After APTMS coating, there is an obvious increase in the density of spherical particles on the APTMS@APC–0.125 surface.

In Figure 2e, the FTIR investigations of APC and APTMS@APC–0.125 are shown. The broad band at 3441 cm^−1^ is the characteristic absorption peak of –OH, corresponding to the dense surface hydroxyl group and adsorbed H_2_O molecules [34,35]. However, the band at 3441 cm^−1^ in the spectra of APC is apparently stronger than that of APTMS@APC–0.125, suggesting that the former could be more hydrophilic. The peak at 1174 cm^−1^ for APC belongs to C–O–C stretching vibrations [14,30,31]. The peak at 1629 cm^−1^ for APC is attributed to –OH bending [35], while 1622 cm^−1^ for APTMS@APC–0.125 is assigned to –OH or –NH_2_ bending [36]. In addition, the peaks at 2930 and 2865 cm^−1^ for APTMS@APC–0.125 are attributed to the C–H stretching vibrations of –CH_3_ and –CH_2_– [35]; those at 1128 and 1025 cm^−1^ can be corresponded to the C–O stretching vibrations and Si–O bond asymmetric stretching vibrations [36,37]; a peak at 775 cm^−1^ is related to Si–C bond stretching vibrations [38]; and a peak at 686 cm^−1^ is assigned to the N–H bending vibration [35]. These peaks suggest that APTMS is successfully grafted onto the APC.

The structure of different samples is investigated via X-ray diffraction (XRD) in Figure 2f. The XRD patterns of both samples exhibit two broad diffraction peaks appearing at 23.5° and 43.4°, corresponding to the (002) and (100) planes of carbon structure, respectively. The most prominent feature in XRD patterns for both the samples is peak (002), which is presumably due to the stacking defect of char crystallites [39]. The (100) slight peak suggests the sp^2^-hybridized arrangement of graphite structure. No other sharp or strong peaks in the XRD spectra were found, which indicates that the APTMS@APC–0.125 is still mainly amorphous after APTMS coating.

Raman spectroscopy (Figure 2g) is an experimental technique which is sensitive to the carbon state; therefore, it can be used to analyze the structure of APCs. The characteristic peaks in the Raman spectrum of carbon materials are mainly D and G peaks, where the D peak is attributed to the disordered or defected sp^3^-bonded carbon, and the G peak originates from the in-plane vibrations of the sp^2^-bonded carbon [40]. The intensity ratio of D and G peaks (*I*_D_/*I*_G_) presents the graphitization degree of carbon materials. The higher the ratio of *I*_D_/*I*_G_, the lower the degree of graphitization and the more disordered and defective the structures [40,41]. It is found that the *I*_D_/*I*_G_ value of APC is about 1.89, which is obviously higher than that on APTMS@APC–0.125 (*I*_D_/*I*_G_ = 1.53), implying that the coating of APTMS decreases the disarray of APC [41]. APC and APTMS@APC–0.125 are mainly composed of amorphous carbon, which is consistent with the XRD results. The above agrees with the results found in a recently published paper [14], illustrating that APTMS coating on the APC obtained by the activation of H_3_PO_4_ is an effective modification method.

### 2.2. Effect of APTMS on Textural Properties and Hydrophobicity

**Figure 3:**
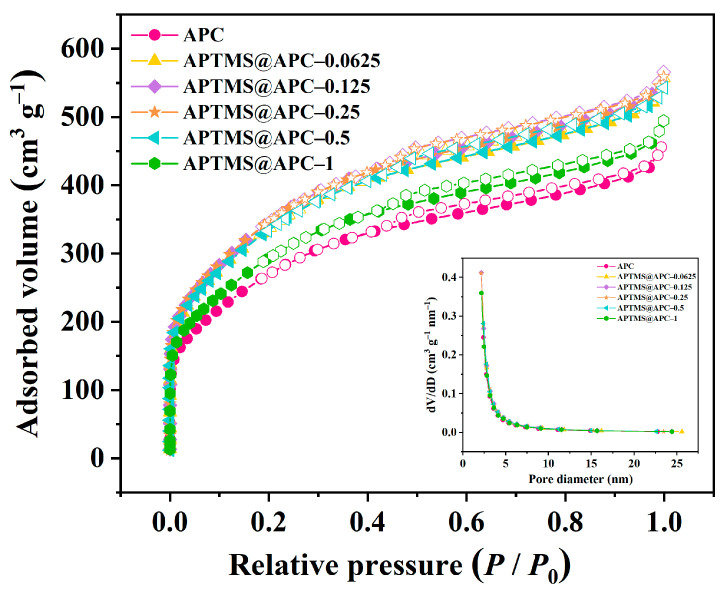
N_2_ adsorption/desorption isotherms and the pore size distribution profiles (inner) of APCs.

The N_2_ adsorption/desorption isotherms and pore size distribution curves of APCs are shown in Figure 3, which are used to analyze their textural properties. Correspondingly, Table 1 lists their textural parameters, including the specific surface area (*S*_BET_), total pore volume (*V*_tot_), mesopore volume (*V*_meso_), micropore volume (*V*_micro_), and average pore size (*D*_aver_), which are calculated from these experimental data. As can be seen, these six APCs presented type I isotherm, first rising steeply at a low relative pressure region (*P*/*P*_0_ < 0.05), and then rising slowly, finally tending to approach a saturation plateau. Furthermore, the BET specific surface areas of APC, APTMS@APC–0.0625, APTMS@APC–0.125, APTMS@APC–0.25, APTMS@APC–0.5, and APTMS@APC–1 are found to be 981, 1208, 1274, 1243, 1206, and 1081 m^2^ g^−1^, respectively. With an increase in APTMS loading, the *S*_BET_ and *V*_tot_ of these samples increase first and then decrease, and APTMS@APC–0.125 has the best texture properties; therefore, it is speculated that it is the best adsorbent. When the APTMS content is low, APTMS is mainly deposited on the outer surface of the sample, so the specific surface area increases gradually with the increase in APTMS concentration. However, with a further increase in the APTMS concentration, APTMS molecules start to aggregate in the pores of APC, blocking the pores and resulting in a decrease in specific surface area.

**Figure 4:**
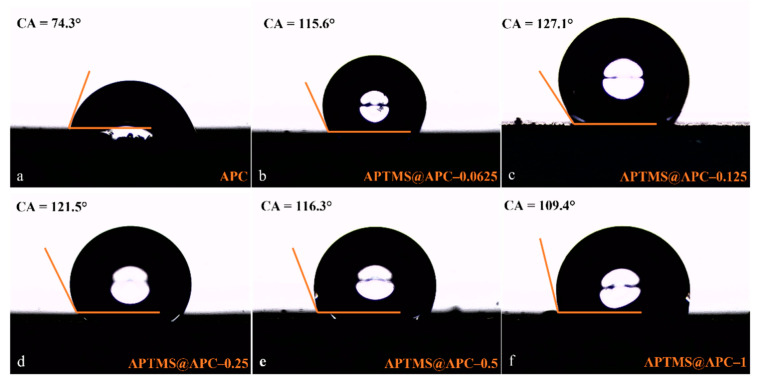
Contact angle of APC (**a**), APTMS@APC–0.0625 (**b**), APTMS@APC–0.125 (**c**), APTMS@APC–0.25 (**d**), APTMS@APC–0.5 (**e**), and APTMS@APC–1 (**f**).

For studying the adsorption process of VMS, porous activated carbons with different degrees of hydrophobicity were prepared via varying mass ratios of APTMS. In our previous article, we already showed that the more hydrophobic the surface, the stronger the adsorption performance [24]. Water contact angle (CA) measurements were performed to determine the hydrophobic properties of APC adsorbents, as shown in Figure 4a–f. We can find that the CA first increases and then decreases with the increase in APTMS. This indicates clearly that the hydrophobicity shows a trend of increasing first and then decreasing, which is identical to the trend in the specific surface area.

**Figure 5:**
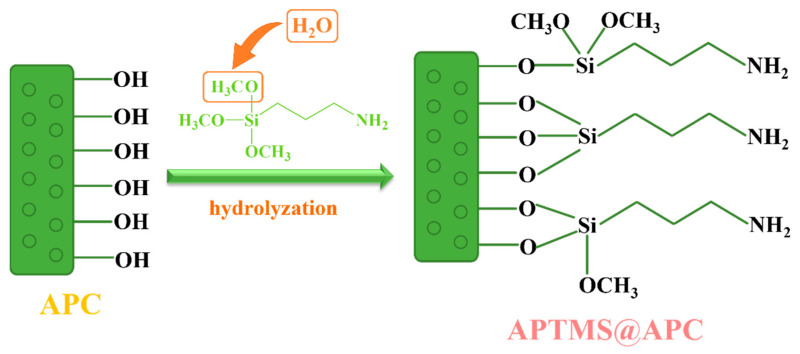
Working mechanism of APTMS@APC adsorbent formation.

Figure 5 illustrates the working mechanisms of APTMS@APC adsorbent formation. There are three possibilities for the mechanism of the connection between APTMS and APC. These results indicate that water can lead to the hydrolysis of APTMS molecules during the silylation reaction, thus condensing with the already present hydroxyl group. APTMS can be bonded to the APC surface by one, two, or three bridged hydroxyl groups depending on the density of –OH groups on the APC surface [35,42]. Also, the bands of –OCH_3_, Si–O, and –NH_2_ are evident in the FTIR spectra (Figure 2e). Furthermore, the increased specific surface area of APTMS@APC may be related to the increased proportion of micropores and mesoporous pores during the silylation reaction process [43,44].

### 2.3. Effect of APCs on Dynamic Adsorption Performance

**Figure 6:**
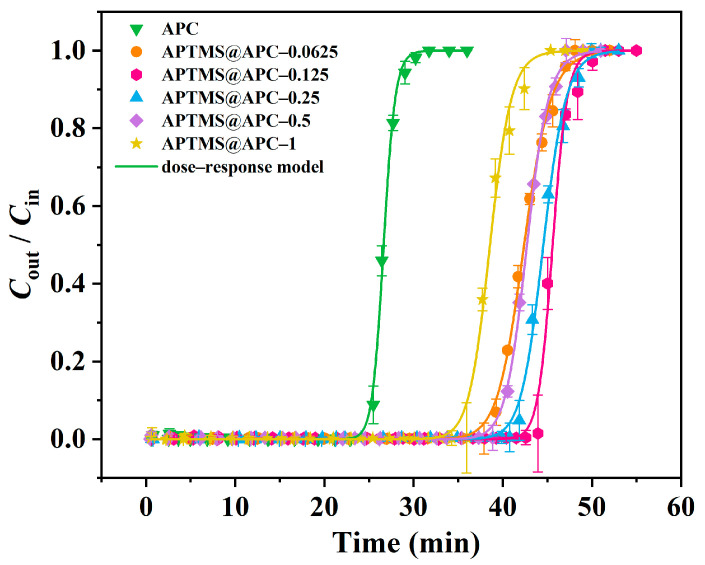
Breakthrough adsorption curves and fitted trajectories of the APCs for L2.

**Figure 7:**
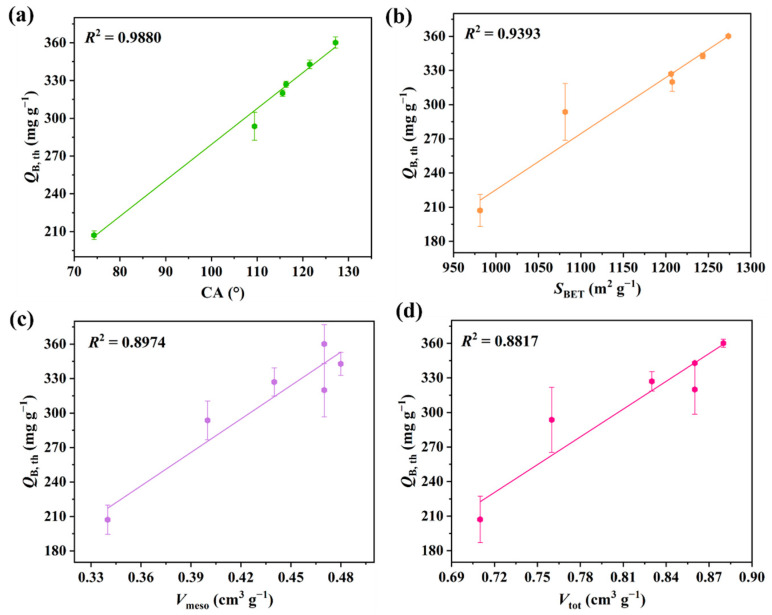
Relationship between *Q*_B,th_ and CA (**a**), *S*_BET_ (**b**), *V*_meso_ (**c**), and *V*_tot_ (**d**) for APTMS@APC–0.125.

Under the conditions of *T* = 25 °C, *C*_in_ = 83.82 mg L^−1^, and *V*_g_ = 50 mL min^−1^, the dot plots of experimental data as well as their dose–response model fitting curves are used to quantitatively analyze the adsorption L2 performance for the APCs, as shown in Figure 6 and Table 2. Each of the six S shapes of the L2 breakthrough adsorption curve can be divided into three stages: plateau; penetration; equilibrium. This behavior is similar to the conventional typical gas–solid adsorption processes, and it can be seen that the dose–response model presents a good fit with these dynamic adsorption data (correlation coefficient *R*^2^ = 0.9957–0.9991). The experimentally measured values (*t*_B_, *Q*_B_, and *Q*_m_) are close to the corresponding theoretical metrics (*t*_B,th_, *Q*_B,th_, and *Q*_m,th_), which are used in our follow-up analyses. As indicated by these results, the adsorption performance of different APCs for L2 from large to small follows the order: APTMS@APC–0.125 > APTMS@APC–0.25 > APTMS@APC–0.5 > APTMS@APC–0.0625 > APTMS@APC–1 > APC. It can be seen that the *t*_B,th_, *Q*_B,th_, and *Q*_m,th_ values of APTMS@APC–0.125 are 43.29 min, 360.1 mg g^−1^, and 380.4 mg g^−1^, respectively, exhibiting the highest adsorption capacity of L2. The above experimental results reveal that the APTMS coating modification can improve the adsorption performance of L2 on the APCs, and with the addition of APTMS, the adsorption capacity increases first and then decreases. When the amount of APTMS added is 0.125% (APTMS mass fraction in water), the adsorption capacity of L2 in APTMS@APC–0.125 reaches the maximum. Interestingly, the trend of L2 adsorption performance is well consistent with the textural and hydrophobicity result. Therefore, to determine the main factors affecting the adsorption capacity of L2, correlational analyses are observed between the L2 adsorption capacity *Q*_B,th_ and CA, *S*_BET_, *V*_meso_, and *V*_tot_, as depicted in Figure 7a–d. The relevant parameters simulated by the y = a + bx equation are shown in Table S2. Positive correlations are all observed between *Q*_B,th_ and CA, *S*_BET_, *V*_meso_, as well as *V*_tot_. The *R*^2^ values for the *Q*_B,th_–CA, *Q*_B,th_–*S*_BET_, *Q*_B,th_–*V*_meso_, and *Q*_B,th_–*V*_tot_ are 0.9880, 0.9393, 0.8974, and 0.8817, respectively. The larger the *R*^2^ value, the better the linear relationship. It can be seen that CA, *S*_BET_, and *V*_meso_ are the main affecting factors for L2 removal; therefore, it can thus be inferred that capillary condensation and the surface’s hydrophobic interactions should be the main adsorption mechanism of L2 on the APTMS@APC–0.125 [24,29].

### 2.4. Effect of Solvents on Adsorption

**Figure 8:**
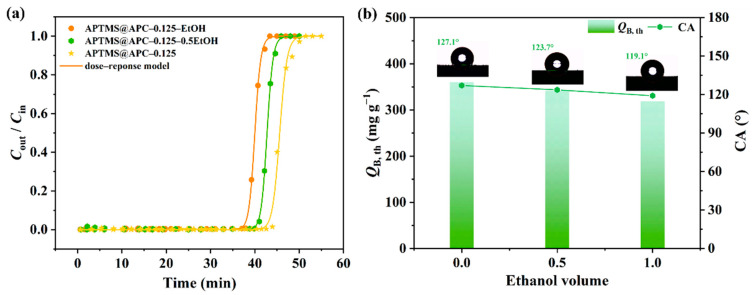
Breakthrough adsorption curves and fitted trajectories of the APCs in different solvents for L2 (**a**) and the trends of *Q*_B,th_ and CA with EtOH volume (**b**).

To examine the effect of solvents on the hydrophobic property of APC, ethanol (EtOH) was used as a disperser solvent of APTMS. The breakthrough adsorption curves and fitted trajectories of the APTMS@APC–0.125 prepared with different solvents are shown in Figure 8a. The *t*_B,th_ of L2 on the three materials show negative correlations with an increase in EtOH volume fractions. In addition, as shown in Figure 8b, the strength of the hydrophobicity decreases (127.1–119.1°) with increasing volume fractions of EtOH. It is speculated that EtOH is a polar protic solvent with hydroxyl groups, and in the case of higher concentrations, these result in a reduction in a dehydrated condensation of Si–OH with APC surfaces and a thinner APTMS coating on APC surfaces [45,46]. Meanwhile, it results in a decreased hydrophobicity of the APTMS@APC–0.125 surface. Therefore, water is the best choice for APTMS dispersion solvent.

### 2.5. Effect of Process Conditions on Adsorption

**Figure 9:**
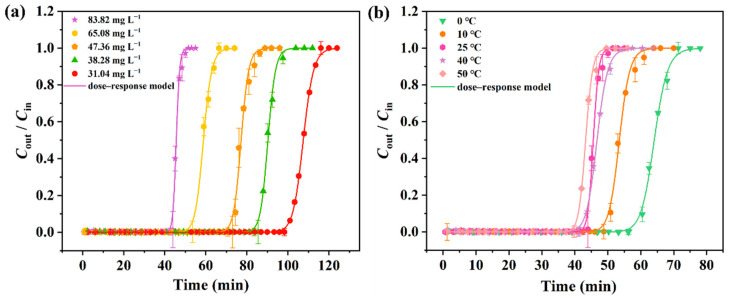
Experimental breakthrough curves and model fitted trajectories for APTMS@APC–0.125 at different inlet concentrations (**a**) and temperatures (**b**).

We explored the influence of different L2 inlet concentrations (Figure 9a, *C*_in_, 31.04–83.82 mg L^−1^) and bed temperatures (Figure 9b, *T*, 0–50 °C) on the adsorption performance of APTMS@APC–0.125 for L2 while keeping the other conditions unchanged. It can be seen in Figure 9 that all the breakthrough curves of L2 show similar S-shaped curves of three clearly characteristic stages, but the curves are shifted to the left with an increase in *C*_in_ and *T*. In addition, the calculated model parameters and the theoretical metrics (*t*_B,th_, *Q*_B,th_, and *Q*_m,th_) based on the experimental data are listed in Table 3. The *Q*_B,th_ is observed to increase upon increasing the concentration and decreasing the temperature, indicating a better adsorption performance of APTMS@APC–0.125 at high concentrations and low temperatures. These results could be due to the exothermic capillary condensation adsorption of L2 in the APTMS@APC–0.125-filled bed [21,29].

### 2.6. Assessment of Regeneration Capacity

**Figure 10:**
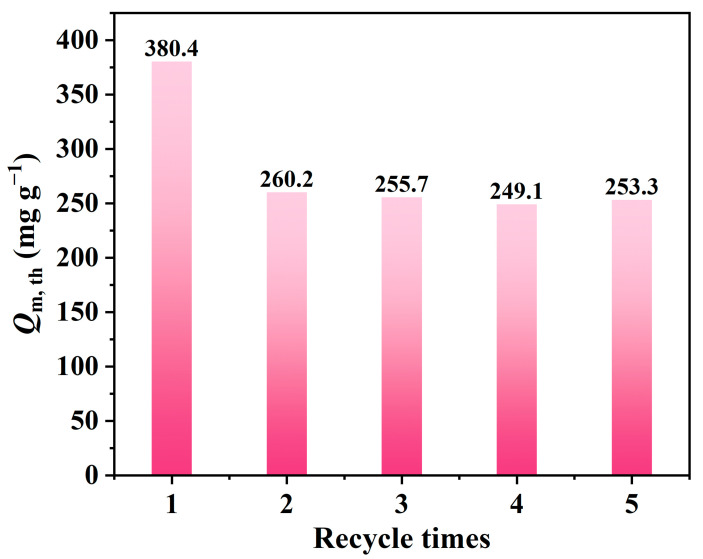
*t*_B,th_ and *Q*_B,th_ of APTMS@APC–0.125 for L2 after each cycle.

APTMS@APC–0.125 after the adsorption of L2 is regenerated after being treated in a 100 °C water bath for 100 min, repeated for five adsorption–desorption cycles. The experimental results of five adsorption/desorption cycles are shown in Figure 10. Although the L2 breakthrough adsorption capacity for APTMS@APC–0.125 after the second regeneration decreased from 380.4 to 253.3 mg g^−1^, the breakthrough adsorption capacity of APTMS@APC–0.125 after the fifth regeneration is still higher than that of the initial APC (223.2 mg g^−1^). From Table 4, the APTMS@APC–0.125 shows the highest adsorption capacity and recycling performance compared with several other carbon materials.

## 3. Materials and Methods

### 3.1. Materials and Chemicals

The coconut shells were purchased from Zhaoqing Chenxing Agriculture Co., Ltd. (Zhaoqing, China). Phosphoric acid (A.R., H_3_PO_4_) was obtained from Tianjin Hengxing Chemical Preparation Co., Ltd. (Tianjin, China). Ethanol (A.R., EtOH) was obtained from Tianjin Yongda Chemical Reagent Co., Ltd. (Tianjin, China). (3–Aminopropyl)trimethoxysilane (97%, APTMS) and hexamethyldisiloxane (99%, L2) were obtained from Aladdin Industrial Corporation (Shanghai, China). Deionized (DI) water were used for the preparation of APCs.

### 3.2. Preparation of APCs

#### 3.2.1. APC Preparation

Activated porous carbon (APC) was prepared by impregnation and calcination. Firstly, 10 g coconut shell (with a particle size less than 425 µm and dried at 110 °C for 2 h) was placed in a flask, followed by pouring 30 g H_3_PO_4_ (A.R.), with the mass ratio of coconut shell to H_3_PO_4_ of 1:3. The mixture were stirred for 30 min to obtain a uniform mixture. The mixture was kept at a constant temperature of 110 °C with drying for 2 h. Subsequently, the mixture was placed in a tube furnace for calcination at 800 °C for 2 h under N_2_ atmosphere (100 mL min^−1^) with a heating rate of 10 °C min^−1^. When the sample was cooled to room temperature, the sample was washed with DI water till it reached neutral, and dried at 110 °C for 2 h to obtain the phosphoric acid-modified APC.

#### 3.2.2. APTMS@APC–x Preparation

APTMS solutions of different mass ratios (0, 0.0625, 0.125, 0.25, 0.5, and 1) were dispersed in 10 mL DI water and underwent sonication for 3 h to become a homogeneous solution. An amount of 1 g of the as-prepared APC was soaked in 10 mL of APTMS dispersion solution with different mass ratios. The solution was oscillated at 120 rpm for 12 h at 25 °C. After filtration, the activated porous carbon pulp was dried at 120 °C under vacuum condition for 24 h. The APTMS@APC–x (x: the mass ratio of APTMS) were stored in a desiccator until required. Then, in order to compare the effect of solvents on the hydrophobicity of APTMS@APC, APTMS (APTMS mass ratio was 0.125) was dispersed into ethanol solutions of different concentrations (the volume ratio of ethanol to deionized water was 0.5 and 1) and the above steps were repeated. The prepared samples were named APTMS@APC–0.125–0.5EtOH and APTMS@APC–0.125–EtOH, respectively.

### 3.3. Characterization

Images were taken using a field emission scanning electron microscope (SEM, Hitachi S4800, Chiyoda City, Japan). The structures were characterized by using a D8 Advance X-ray diffractometer equipped with Cu Kα radiation (XRD, λ  =  0.154 nm, Bruker, Bremen, Germany). Using KBr pellets, FTIR spectroscopy was performed by using an FTIR spectrometer (IR Tracer–100, Shi-madzu, Nagoya, Japan) in the region of 4000~500 cm^−1^. Raman spectroscopy measurements were carried out by a Raman spectrometer (XploRA PLUS, Horiba, Japan) using a 514 nm laser. N_2_ adsorption–desorption isotherms of APCs were obtained at 77 K using a Kubo × 1000 surface area and pore size analyzer (Beijing Builder Co., Ltd., Beijing, China). At room temperature, the water contact angle of the APCs was measured using the JY-PHb contact angle system (Chengde Jinhe Co., Ltd., Chengde, China) to characterize the wettability of the APCs’ surface.

### 3.4. Dynamic Adsorption Tests of L2

The removal performance of APTMS@APC–x for L2 gas stream was measured using a fixed-bed dynamic adsorption setup. The feasibility and specific manipulations of such an approach have been reported in detail in our previous work [15,21]. For each test, 0.50 g of APTMS@APC–x was used at 25 °C, an L2 inlet concentration of 83.82 mg L^−1^, and a gas flow rate of 50 mL min^−1^. The adsorbent’s adsorption performance was assessed using breakthrough curves, which were drawn by plotting *C*_out, t_/*C*_in_ with *t*. We employed the following three metrics to evaluate the adsorbent performance: (I) the breakthrough time (*t*_B_, defined as the time when *C*_out, t_/*C*_in_ ≈ 0.05, min); (II) the breakthrough adsorption capacity (*Q*_B_, represented the adsorption capacity at time *t*_B_, mg g^−1^); and (III) the saturated adsorption capacity (*Q*_m_, defined as the adsorption capacity when *C*_out, t_/*C*_in_ ≈ 1, mg g^−1^). Generally, the *Q*_B_ and *Q*_m_ values for an independent adsorption test were found using Equation (2):

(2) $$Q_{t}=\frac{V_{g}C_{\mathrm{in}}}{m}\int_{0}^{t}(1-\frac{C_{\mathrm{out},t}}{C_{\mathrm{in}}})dt$$

where *V*_g_ is the flow rate of the L2 gas (L min^−1^); *m* is the mass of the adsorbent (g); *C*_in_ is the inlet concentration at 0 min (mg L^−1^); *C*_out, t_ is the outlet concentration (mg L^−1^).

The measured dynamic data of L2 gas can be predicted via dose–response model, which can better analyze the dynamic adsorption behavior of L2 in the adsorbent’s fixed-bed column, and the theoretical *t*_B,th_, *Q*_B,th_, and *Q*_m,th_ can be calculated via Equation (3):

(3) $$\frac{C_{\mathrm{out},t}}{C_{\mathrm{in}}}=1-\frac{1}{1+{\left(\frac{C_{\mathrm{in}}\times V_{g}\times t}{q_{0}\times m}\right)}^{a}}$$

where *q*_0_ and a are the dose–response constants of the model.

### 3.5. Regeneration of Spent APCs

When the adsorption of APTMS@APC–0.125 was saturated, the adsorption tubes were placed in a water bath (100 °C) and they were blown with 100 mL min^−1^ N_2_ for 100 min. Five consecutive adsorption/desorption cycles were repeated in the same manner.

## 4. Conclusions

In this study, a novel hydrophobic APTMS@APC adsorbent was developed via a grafting of APTMS functional group on the surface of a H_3_PO_4_-modified APC for greater adsorption capacity for L2. Compared with NaOH modification, this method avoided the corrosion of the quartz tube and improved the hydrophobic, specific surface, mesopore volume, micropore volume, and total pore volume of the APC, with the APTMS@APC–0.125 exhibiting the highest hydrophobic (CA = 127.1°), *S*_BET_ (1274 m^2^ g^−1^), *V*_tot_ (0.88 cm^3^ g^−1^), *V*_meso_ (0.47 cm^3^ g^−1^), and *V*_micro_ (0.41 cm^3^ g^−1^). After hydrophobic modification, the adsorption performance of APTMS@APC for L2 is obviously improved, with the APTMS@APC–0.125 possessing the highest *t*_B,th_ (43.29 min) and *Q*_B,th_ (360.1 mg g^−1^) at 25 °C and the breakthrough adsorption behavior is well described by the dose–response model. There was a high positive correlation among four groups of *Q*_B,th_–CA, *Q*_B,th_–*S*_BET_, *Q*_B,th_–*V*_meso_, and *Q*_B,th_–*V*_tot_, for the as-prepared APTMS@APC–0.125, indicating that the L2 adsorption mechanism followed capillary condensation and the surface’s hydrophobic interactions. Comprehensive analysis of the process conditions revealed that a decrease in the bed temperature and an increase in the inlet concentration could enhance the L2 adsorption capacity for the APTMS@APC–0.125. After five adsorption–desorption cycles, the adsorption capacity of APTMS@APC–0.125 for L2 was still higher than the initial adsorption capacity of APC and other carbon materials. Consequently, this as-prepared APTMS@APC material with hydrophobic and high specific surface area, will be a satisfactory adsorbent to remove the VMS from biogas in industrial applications.

## Figures and Tables

**Table 1:** The textural properties of APCs.

Samples	*S*_BET_/m^2^ g^−1^	*V*_tot_/cm^3^ g^−1^	*V*_meso_/cm^3^ g^−1 a^	*V*_micro_/cm^3^ g^−1^	*D*_aver_/nm	CA/°
APC	981	0.71	0.34	0.37	1.44	74.3
APTMS@APC–0.0625	1208	0.86	0.47	0.39	1.43	115.6
APTMS@APC–0.125	1274	0.88	0.47	0.41	1.37	127.1
APTMS@APC–0.25	1243	0.86	0.48	0.38	1.39	121.5
APTMS@APC–0.5	1206	0.83	0.44	0.39	1.39	116.3
APTMS@APC–1	1081	0.76	0.40	0.36	1.41	109.4

^a^ *V*_meso_ = *V*_tot_ − *V*_micro_.

**Table 2:** Adsorption parameters of APCs for L2.

Adsorbents	Experimental			Dose–Response Model						
	*t*_B_/ min	*Q*_B_/ mg g^−1^	*Q*_m_/ mg g^−1^	*t*_B,th_/ min	*Q*_B,th_/ mg g^−1^	*Q*_m,th_/ mg g^−1^	*q*_0_	*a*	*R* ^2^	Standard Deviation
APC	24.25	200.7	223.2	24.83	207.2	223.2	0.2233	41.24	0.9987	0.01340
APTMS@APC–0.0625	38.88	322.8	356.3	38.19	320.0	357.6	0.3553	28.12	0.9984	0.01359
APTMS@APC–0.125	44.08	361.8	385.4	43.29	360.1	380.4	0.3822	55.57	0.9957	0.02024
APTMS@APC–0.25	41.96	346.9	375.9	41.00	342.8	374.3	0.3734	35.23	0.9978	0.01564
APTMS@APC–0.5	39.65	326.1	353.4	39.32	327.0	356.9	0.3580	35.12	0.9991	0.01021
APTMS@APC–1	36.18	299.5	332.8	35.18	293.7	323.5	0.3233	32.27	0.9954	0.02250

**Table 3:** Adsorption parameters of APTMS@APC–0.125 for L2 at different process conditions.

Term	Value	*t*_B,th_/ min	*Q*_B,th_/ mg g^−1^	*Q*_m,th_/ mg g^−1^	*q*_0_	*a*	*R* ^2^	Standard Deviation
*T*/°C	0	58.42	480.3	529.4	0.5372	31.74	0.9977	0.01409
	10	49.08	402.1	440.2	0.4484	33.74	0.9967	0.02017
	25	43.29	360.1	380.4	0.3822	55.57	0.9958	0.02024
	40	42.15	346.0	385.5	0.3916	28.87	0.9993	0.00861
	50	40.25	329.7	355.4	0.3626	40.96	0.9978	0.01665
*C*_in_/mg L^−1^	83.82	43.29	360.1	380.4	0.3822	55.57	0.9958	0.02024
	65.08	53.19	338.4	376.2	0.4932	29.09	0.9975	0.01703
	47.36	75.00	333.5	359.4	0.6471	42.04	0.9932	0.02854
	38.28	85.07	320.5	341.6	0.7568	48.86	0.9977	0.01334
	31.04	100.40	307.0	330.3	0.9010	42.82	0.9995	0.00680

**Table 4:** Carbon materials with different adsorption and recycling capacities for siloxanes.

Adsorbent	Adsorbed Gas	*Q*_m_, mg g^−1^	Regeneration Method	*Q*_m_, mg g^−1 a^	Reference
AC	D4	526	Oxidation with H_2_O_2_ and O_3_	210.4	[47]
AC	D3	60	Heating at 100–200 °C	30	[48]
AC	L2	100	Four-step heating treatment at 160 °C	70–80	[49]
rGOA–200	L2	32	Heating at 80 °C	32	[29]
APTMS@APC–0.125	L2	380.4	Heating at 100 °C	253.3	This work

^a^ The *Q*_m_ value after multiple adsorption/desorption cycles.

## Data Availability

Data are contained within the article. The data presented in this study are available.

## Supplementary Materials

The following supporting information can be downloaded at: https://www.mdpi.com/article/10.3390/molecules28186493/s1, Table S1: Different preparation methods, textural parameters, and performances of porous absorbent materials for siloxane removal; Table S2: The statistical parameters of curves between *Q*_B,th_–CA, *Q*_B,th_–*S*_BET_, *Q*_B,th_–*V*_meso_, and *Q*_B,th_–*V*_tot_ for the APTMS@APC–0.125.
