# Supplementary material for: Removal of Hexamethyldisiloxane via a Novel Hydrophobic (3–Aminopropyl)Trimethoxysilane-Modified Activated Porous Carbon
Source: Molecules. 2023 Sep 7;28(18):6493. doi: 10.3390/molecules28186493 (PMC10535671; doi:10.3390/molecules28186493)
Supplement: Supplementary file 1 [file molecules-28-06493-s001.zip › molecules-2553389-supplementary.pdf]

Supplementary information

Removal of Hexamethyldisiloxane by a Novel Hydrophobic APTMS–Modified Activated Porous Carbon

Siqi Lv [1], Yingrun Wang [1], Yanhui Zheng [1,2,*] and Zichuan Ma [1,*]

[1] Hebei Key Laboratory of Inorganic Nano–Materials, College of Chemistry and Material Sciences, Hebei Normal University, Shijiazhuang 050024, China
[2] School of Chemical Engineering, Shijiazhuang University, Shijiazhuang 050035, China

Table S1. Different preparation methods, textural parameters, and performances of porous absorbent materials for siloxane removal

Adsorbent	Origin	Modified method	S_{BET}, $m^2\ g^{-1}$	V_{tot}, $cm^3\ g^{-1}$	Adsorbed gas	Q, mg g^{-1}	Ref.
APC–4	Coconut shells	NaOH	2551	1.30	L2	894.7	[14]
AC–8	Commercial	/	1573	0.92	D4	224.6	[17]
AC2	Lignocellulosic waste	K_2CO_3	1668	0.70	L2<br>D4	438.0<br>512.0	[19]
MMSG	Silica gel	PEI[a]	539	0.73	L2	367.1	[20]
MFS2	Silica gel	MTS[b]	1261	1.03	L2	315.4	[21]
UCT–15[c]	Tetraethylorthosilicate and aluminum nitrate	/	533	0.26	D4	105.0	[22]
RPA	Silica gel	PAA[d]	120	/	D5	21.6	[23]
rGOA–2	IGGO[e]	CA[f]	582	2.43	L2	188.3	[15]
U–rGOA	IGGO[e]	Urea	168	0.74	L2	83.9	[24]
APTMS@APC–0.125	Coconut shells	H_3PO_4 and APTMS	1274	0.88	L2	380.4	This work

[a] Polyethylene imine;
[b] Methyltrimethoxymethylsilane;
[c] Mesoporous aluminosilicate;
[d] Polyacrylic acid
[e] Industrial grade multilayer graphene oxide;
[f] Citric acid.

Table S2. The statistical parameters of curves between $Q_{B, th}$ – CA, $Q_{B, th}$ – S_{BET}, $Q_{B, th}$ – V_{meso}, and $Q_{B, th}$ – V_{tot} for the APTMS@APC–0.125

Statistical parameters		$Q_{B, th}$ – CA	$Q_{B, th}$ – S_{BET}	$Q_{B, th}$ – V_{meso}	$Q_{B, th}$ – V_{tot}
Number of Points		6	6	6	6
Equation		y = a + bx			
Residual Sum of Squares		178.4943	898.0011	1518.9473	1751.6294
Standard deviation		5.9749	13.4015	17.4296	18.717
R–Square		0.9880	0.9393	0.8974	0.8817
Intercept	Value	–10.0785	–242.7953	–103.0703	–313.1645
	Standard Error	17.8034	70.3012	70.0219	114.1675
Slope	Value	2.8772	0.4730	949.6968	761.1790
	Standard Error	0.1589	0.0601	160.5428	139.4050
